# Evaluation of the Association between Smoking and Tonsillolith Characteristics Using Cone-Beam Computed Tomography

**DOI:** 10.1159/mpp/adpag014

**Published:** 2026-09-02

**Authors:** Ali Rasat, Sevcihan Günen Yılmaz

**Affiliations:** a Department of Oral and Maxillofacial Radiology, Faculty of Dentistry, Burdur Mehmet Akif Ersoy University, Burdur, Türkiye; b Department of Oral and Maxillofacial Radiology, Faculty of Dentistry, Akdeniz University, Antalya, Türkiye

**Keywords:** Cone-beam computed tomography, Tonsillolith, Smoking

## Abstract

**Objective:**

Tonsilloliths are calcified concretions within tonsillar crypts whose relationship with smoking remains poorly understood. This study investigated the association between smoking and tonsillolith characteristics using cone-beam computed tomography (CBCT).

**Subject and Methods:**

This retrospective cross-sectional study evaluated 416 dental CBCT scans obtained between 2021 and 2023. Scans were evaluated for the presence, size, number, laterality, and anatomical localization of tonsilloliths by a single oral and maxillofacial radiologist, with a second radiologist consulted in cases of diagnostic uncertainty. Demographic characteristics and smoking status were obtained from medical records. Statistical analyses included the chi-square or Fisher’s exact test, independent-samples *t* test or Mann-Whitney U test, and one-way ANOVA with Bonferroni post hoc correction (α = 0.05).

**Results:**

Tonsilloliths were detected in 29.6% of participants (123/416). Smoking was not significantly associated with tonsillolith prevalence, size, or number (*p* > 0.05). Among patients with tonsilloliths, smokers showed significantly higher frequencies of unilateral (*p* = 0.041) and lingual (*p* = 0.036) tonsilloliths than non-smokers. Age was significantly associated with both prevalence and size (*p* < 0.001 and *p* = 0.037, respectively), with higher prevalence in older groups. No significant sex differences were found.

**Conclusion:**

Smoking was not associated with the overall prevalence, size, or number of tonsilloliths, but unilateral and lingual tonsilloliths were more frequent among smokers. Age was the strongest factor associated with tonsillolith formation, exerting a greater influence than smoking. Further prospective, multicenter studies with detailed smoking exposure data are needed to validate these findings.

**Highlights of the Study**

- Tonsillolith prevalence detected on cone-beam computed tomography was 29.6%, and smoking did not significantly affect overall prevalence, size, or number of tonsilloliths.
- Smokers showed significantly higher rates of unilateral and lingual tonsillolith localization compared with non-smokers.
- Age was the strongest factor associated with tonsillolith prevalence and size, with higher rates observed in older age groups.

## Introduction

Soft tissue calcifications in the head and neck region may primarily result from physiological or pathological mineralization caused by irregular calcium phosphate deposits, known as heterotopic calcification [1]. The tonsils are lymphoid tissues located on the lateral walls of the oropharynx on the right and left sides. Tonsilloliths are calcified concretions that form within the tonsillar crypts, often associated with chronic inflammation, accumulation of debris, bacteria, and epithelial cells [2]. Although rare in children, they are frequently observed in adults. Small ones are asymptomatic; large ones can cause symptoms such as pain, difficulty swallowing, foreign body sensation, and halitosis [3, 4]. Tonsilloliths may be incidentally detected during examination of a panoramic radiograph taken for any purpose, where they usually appear as radiopaque areas superimposed on the mid-ramus region [5]. Information about the shape, number, size, and location of tonsilloliths can be determined using cone-beam computed tomography (CBCT). Tonsilloliths are more common in adults and in individuals with recurrent chronic tonsillitis as repeated inflammation facilitates the accumulation of debris in the tonsillar crypts. Their etiology is multifactorial and may be associated with upper respiratory tract inflammation, dry mouth, changes in the microbial flora, insufficient saliva secretion, and poor oral hygiene [6–8]. Smoking causes chronic hypoxia in vascular structures by reducing oxygen utilization and increasing carbon monoxide levels, while also altering oral flora and decreasing saliva flow [9, 10].

Although tonsilloliths are frequently detected as incidental findings in adult CBCT examinations, the influence of smoking on their prevalence and radiological characteristics has not been clearly defined, and the available evidence regarding this association remains limited and largely indirect. To our knowledge, no previous study has evaluated the effect of smoking on both the prevalence and localization patterns of tonsilloliths using CBCT. Therefore, this study aimed to investigate the association between smoking and tonsillolith prevalence, size, number, laterality, and anatomical localization.

## Subject and Methods

### Sample Size Calculation

An a priori sample size calculation was performed using G*Power software (version 3.1.9.4; Heinrich-Heine-Universität Düsseldorf, Germany) based on the primary objective of comparing the prevalence of tonsilloliths between smokers and non-smokers. Assuming a medium effect size (w = 0.50), a two-tailed α level of 0.05, and a statistical power of 0.95, the minimum required sample size was 210 participants.

### Study Design

This retrospective study examined CBCT images taken for different dental indications, such as implant planning, extraction of impacted teeth, and orthognathic surgery, at the Akdeniz University Faculty of Dentistry, Department of Oral and Maxillofacial Radiology, between 2021 and 2023. A total of 439 CBCT images were evaluated for eligibility. Only CBCT images that captured the relevant anatomical regions and exhibited optimal diagnostic quality were included; scans encompassing the oropharynx and nasopharynx were reviewed for calcifications within the tonsillar region. Poor-quality images or scans with an unsuitable field of view for detecting tonsilloliths were excluded. Of the images evaluated, 23 did not meet the inclusion criteria and were excluded. After data verification, the final analytic sample comprised 416 patients, with ages ranging from 18 to 82 years (mean 44.3 ± 12.5 years). For descriptive and comparative purposes, the study population was divided into four age groups (18–35, 36–50, 51–65, and ≥66 years) to ensure adequate sample size within each category and to facilitate comparison with previous studies that use similar age bands.

All CBCT images were obtained using the Veraview X800 (J. Morita Mfg. Corp., Kyoto, Japan) according to the manufacturer’s standard parameters (FOV: 15 × 14 cm; 4.8 mA; 99 kVp; 35.8 s exposure). Scans were evaluated in 1-mm-thick coronal, axial, and sagittal slices using i-Dixel 2.0 three-dimensional imaging software (J. Morita, Kyoto, Japan) on a 22-inch full HD (1,920 × 1,080) monitor in a dimly lit room, following standard positioning protocols. Only images with a 15 × 14 cm region of interest were used to ensure standardization. For each identified tonsillolith, age group (18–35, 36–50, 51–65, ≥66 years), sex, laterality, size (mm), number (single or multiple), smoking status, daily cigarette consumption (0–10, 11–20, ≥21), and anatomical localization (palatine/lingual/both) were recorded (Fig. 1). Smoking status and daily cigarette consumption data were obtained from patients' medical records. The maximum diameter of each tonsillolith was measured in the plane in which it appeared largest, using axial, coronal, and sagittal reconstructions.

**Fig. 1.**
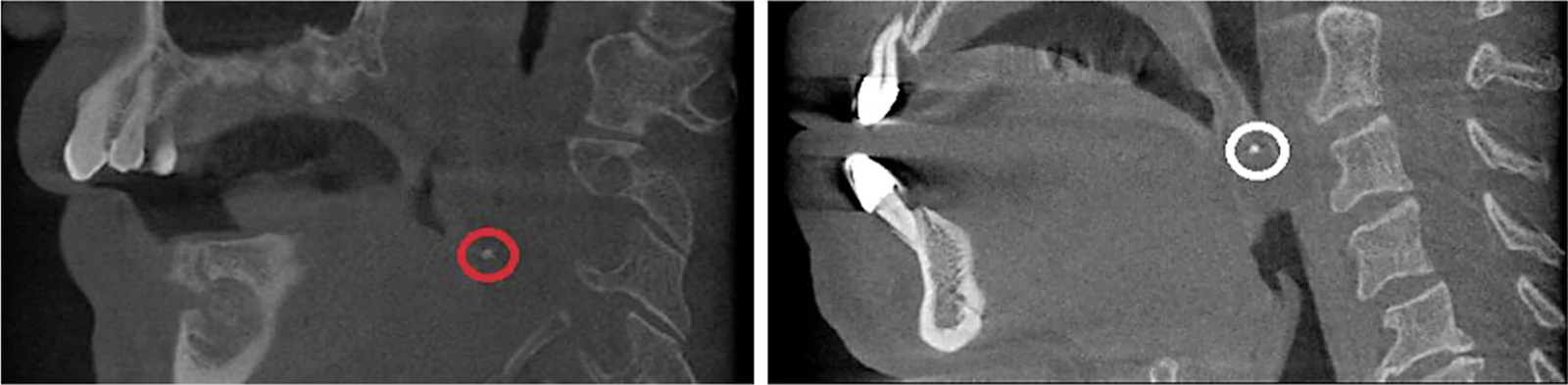
Sagittal CBCT images demonstrating tonsilloliths. Red circle: lingual tonsillolith; white circle: palatine tonsillolith.

### Tonsillolith Characterization

For each identified tonsillolith, the following parameters were recorded.

(a) Presence: binary classification (present or absent).
(b) Size: maximum diameter measured using electronic calipers in the plane where the tonsillolith appeared largest on axial, coronal, or sagittal reconstructions, recorded to the nearest 0.1 mm.
(c) Number: single (one tonsillolith per patient) or multiple (two or more tonsilloliths per patient).
(d) Laterality: unilateral (tonsillolith(s) detected on one side only) or bilateral (tonsillolith(s) detected on both sides). For lingual tonsilloliths, which arise within the midline lingual tonsillar tissue rather than paired anatomical structures, laterality was operationally defined according to the distribution of calcifications relative to the midline on axial CBCT images [11]. Calcifications confined to one side of the midline were classified as unilateral, whereas those present on both sides of the midline were classified as bilateral.
(e) Anatomical localization: palatine (within the palatine tonsil), lingual (within the lingual tonsil), or both (tonsilloliths present in both anatomical locations).

### Observer and Reliability Assessment

All CBCT scans were primarily evaluated by a single oral and maxillofacial radiologist (A.R.) with 5 years of experience in CBCT interpretation, who was blinded to the patients’ smoking status throughout image assessment. A second experienced oral and maxillofacial radiologist (S.G.Y., 15 years of experience) was consulted only in cases of diagnostic uncertainty to reach a consensus diagnosis. Therefore, the image assessment was not performed independently by two observers, and interobserver reliability was not calculated.

To assess intraobserver reliability, 40 randomly selected CBCT scans (9.6% of the total sample) were re-evaluated by the same observer after a 2-week washout period, with a maximum of 20 patients assessed per day under standardized viewing conditions. Cohen’s kappa coefficient was calculated for categorical variables (presence, laterality, and localization), and the intraclass correlation coefficient was calculated for the continuous variable (size). Intraobserver agreement was excellent (κ = 0.96, 95% confidence interval [CI]: 0.91–1.00 for presence; intraclass correlation coefficient = 0.94, 95% CI: 0.89–0.97 for size).

### Statistical Analysis

All statistical analyses were performed using SPSS Statistics version 23.0 (IBM Corporation, Armonk, NY, USA). A significance level of α = 0.05 (two-tailed) was applied for all tests. Continuous variables were first assessed for normality using the Shapiro-Wilk test and for homogeneity of variance using Levene’s test. Normally distributed continuous variables were presented as mean ± standard deviation, whereas non-normally distributed variables were presented as median and interquartile range. Categorical variables were summarized as frequencies and percentages, and where appropriate, 95% CIs were calculated.

For comparative analyses between groups, different statistical tests were applied depending on the type and distribution of variables. Categorical variables were analyzed using the chi-square test or Fisher’s exact test when expected cell frequencies were less than five. Continuous variables comparing two groups were analyzed using the independent samples *t* test for normally distributed data or the Mann-Whitney U test for non-normally distributed data. For comparisons involving more than two groups, one-way ANOVA was used for normally distributed variables and the Kruskal-Wallis test for non-normally distributed variables. When overall tests involving multiple groups (such as age categories or smoking intensity) were statistically significant, post hoc pairwise comparisons were performed using the Bonferroni correction to control for type I error inflation.

## Results

A total of 416 cases were included in the study. Participants’ ages ranged from 18 to 82 years, with a mean age of 44.3 ± 12.5 years. The distribution across age categories was 18–35 years (*n* = 108, 26.0%), 36–50 years (*n* = 154, 37.0%), 51–65 years (*n* = 119, 28.7%), and ≥66 years (*n* = 35, 8.4%). A total of 47.3% (*n* = 197) of the cases were male, and 52.7% (*n* = 219) were female participants. Tonsilloliths were detected in 123 cases (29.6%), while 293 cases (70.4%) had no tonsilloliths. Demographic characteristics are summarized in Table 1. The prevalence of tonsilloliths was 31.3% (*n* = 62/198) in the group of smokers and 28.0% (*n* = 61/218) in the group of non-smokers, as shown in Figure 2. This difference was not statistically significant (*p* = 0.505). The correlation between smoking intensity (daily cigarette consumption groups: 0–10, 11–20, ≥21) and the occurrence of tonsilloliths was not statistically significant (*p* = 0.713). Therefore, smoking or daily amount did not affect the overall prevalence of tonsilloliths.

**Table 1.** Demographic characteristics of the study population

Characteristic	Overall (*n* = 416)	Smokers (*n* = 198)	Non-smokers (*n* = 218)
Age (years)			
Mean±SD	44.3±12.5	45.1±11.8	43.6±13.1
Range	18–82	20–79	18–82
Median (IQR)	45 (35–53)	46 (37–54)	44 (34–52)
Age group, *n* (%)			
18–35 years	85 (20.4)	38 (19.2)	47 (21.6)
36–50 years	156 (37.5)	72 (36.4)	84 (38.5)
51–65 years	133 (32.0)	68 (34.3)	65 (29.8)
≥66 years	42 (10.1)	20 (10.1)	22 (10.1)
Sex, *n* (%)			
Male	197 (47.3)	102 (51.5)	95 (43.6)
Female	219 (52.7)	96 (48.5)	123 (56.4)
Smoking status, *n* (%)			
Non-smoker	218 (52.4)	–	218 (100.0)
Current smoker	198 (47.6)	198 (100.0)	–
Smoking intensity (smokers only)			
Light (<10 cig/day)	–	67 (33.8)	–
Moderate (10–20 cig/day)	–	89 (44.9)	–
Heavy (>20 cig/day)	–	42 (21.2)	–
Tonsillolith prevalence			
Present, *n* (%)	123 (29.6)	62 (31.3)	61 (28.0)
Absent, *n* (%)	293 (70.4)	136 (68.7)	157 (72.0)

SD, standard deviation; IQR, interquartile range; cig, cigarettes; *n*, number of participants.

**Fig. 2.**
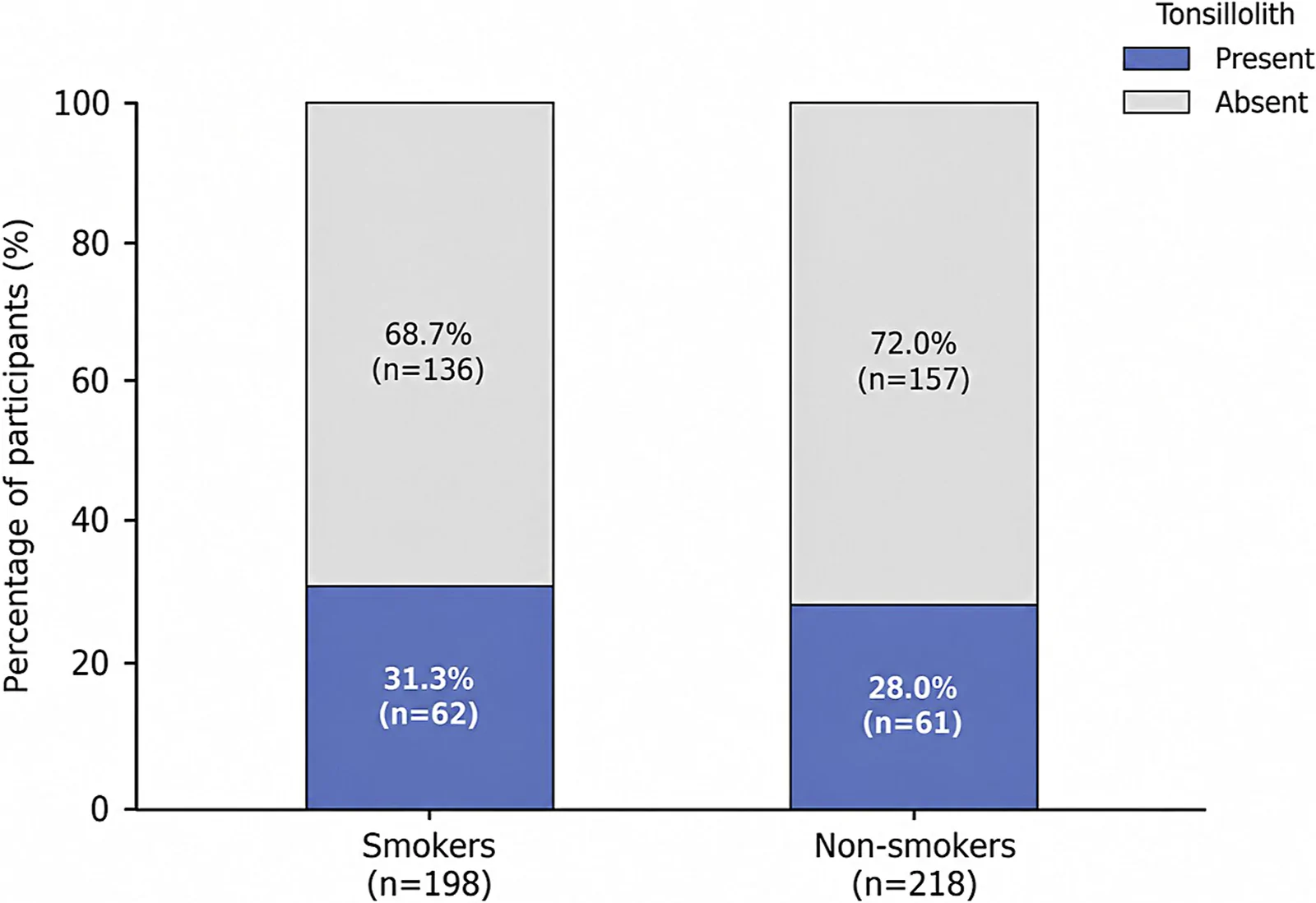
Distribution of tonsillolith presence according to smoking status.

In 123 cases with tonsilloliths, the maximum diameter of the stones was 2.14 ± 1.19 mm on average. There was no significant difference in size between smokers and non-smokers (2.23 ± 1.08) (*p* = 0.644). Similarly, no significant difference was found when comparing groups based on the number of cigarettes smoked per day (*p* = 0.323). In terms of the number of tonsilloliths, 54.5% of cases contained a single tonsillolith, while 45.5% contained multiple tonsilloliths. No significant relationship was found between smoking status (*p* = 0.323) and number of cigarettes smoked (*p* = 0.701) in terms of number distribution.

Regarding anatomical localization, 15.1% of the participants (*n* = 63) had lingual tonsilloliths, 8.9% (*n* = 37) had palatine tonsilloliths, and 5.5% (*n* = 23) had both lingual and palatine tonsilloliths. Among individuals with tonsilloliths, the distribution of tonsillolith localization (palatine/lingual/palatine-lingual) differed significantly between smokers and non-smokers (*p* = 0.036). Specifically, lingual tonsilloliths were more common in smokers (*n* = 36, 58.1% of smokers with tonsilloliths) compared to non-smokers (*n* = 27, 42.6% of non-smokers with tonsilloliths). Conversely, palatine tonsilloliths were more frequent in non-smokers (*n* = 25, 39.3%) than in smokers (*n* = 12, 19.4%). Furthermore, laterality analysis showed a statistically significant difference between smokers and non-smokers (*p* = 0.041). Unilateral tonsilloliths were significantly more common in the smoking group (*n* = 47, 75.8%) compared to the non-smoking group, as shown in Table 2 (*n* = 35, 55.7%).

**Table 2.** Comparison of tonsillolith characteristics between smokers and non-smokers

Variable	Smokers (*n* = 62)	Non-smokers (*n* = 61)	*p* value
Tonsilloliths, *n*	62	61	0.505
Tonsillolith size, mm (mean±SD)	2.14±1.19	2.23±1.08	0.644
Number (single/multiple)	36/26	29/32	0.323
Laterality (unilateral/bilateral)	47/15	35/26	**0.041**
Localization (palatine/lingual/both)	12/36/14	25/27/9	**0.036**

*p* values were calculated using the chi-square test or Fisher’s exact test for categorical variables and the Mann-Whitney U test for tonsillolith size. *n*, number of participants; mm, millimeters; SD, standard deviation.

The presence of tonsilloliths showed statistically significant differences between age groups (*p* < 0.001). The tonsillolith rate was 16.2% for people aged 18 to 35, 29.0% for people aged 36 to 50, 37.1% for people aged 51 to 65, and 56.8% for people aged 66 and older (Fig. 3.). This result indicates that the frequency of tonsilloliths increases with age. The prevalence of tonsilloliths was 30.2% in men and 27.4% in women, indicating no significant difference (*p* = 0.515). The mean size of tonsilloliths in cases with tonsilloliths showed a significant difference according to age groups (*p* = 0.037). A tendency for stone size to increase with age was observed. No difference in size was found between genders (*p* = 0.443).

**Fig. 3.**
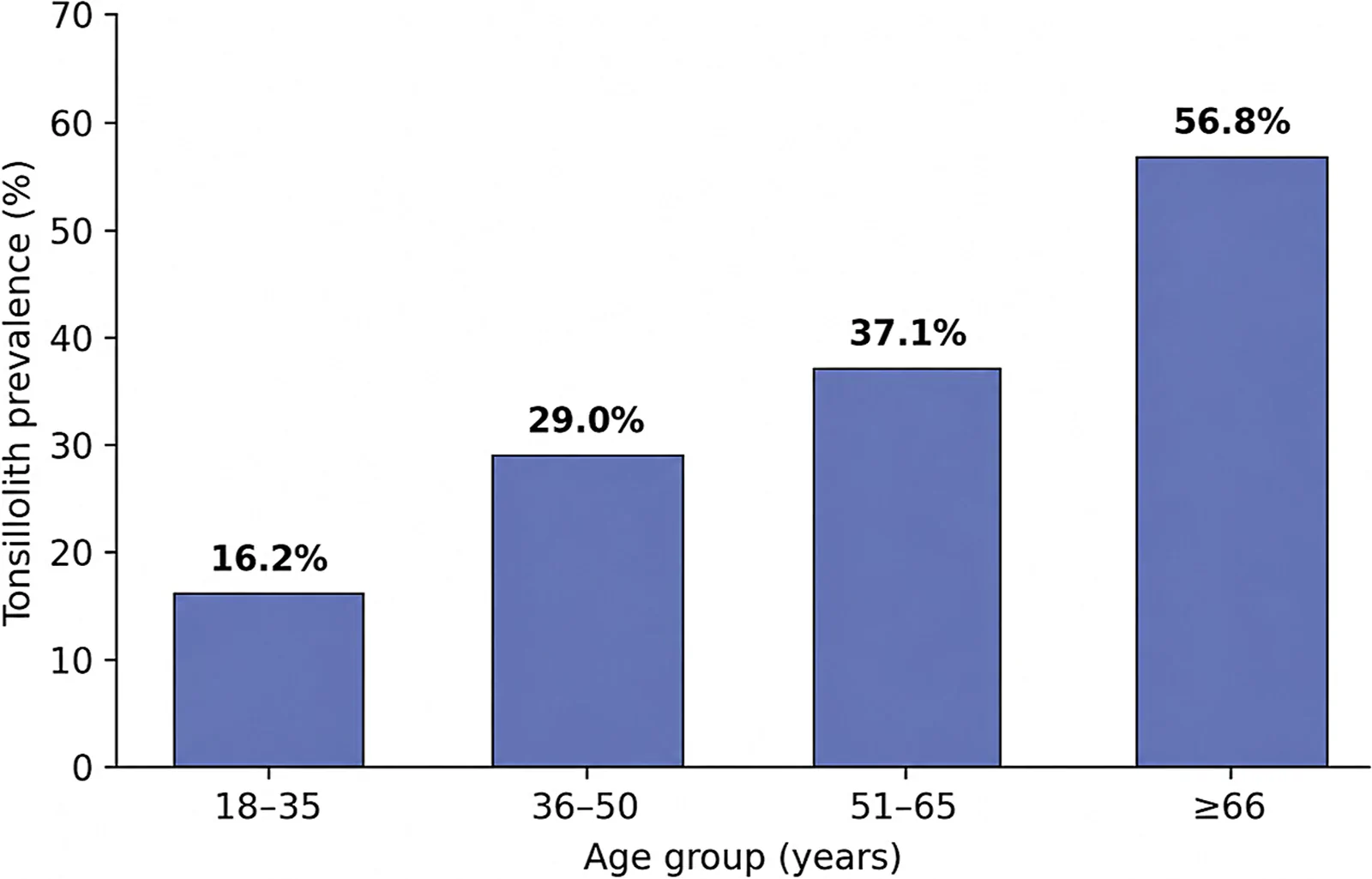
Tonsillolith prevalence by age group.

## Discussion

This study aimed to investigate the relationship between the presence and characteristics of tonsilloliths (size, number, laterality, and anatomical localization) and demographic and behavioral factors, including age, sex, and smoking (smoking status and daily cigarette consumption). The findings indicated that both the presence and size of tonsilloliths were significantly associated with age (*p* < 0.001 and *p* = 0.037, respectively), with the highest prevalence (56.8%) and the largest mean size (2.74 mm) observed in the ≥66-year age group. Smoking was not associated with the overall prevalence, size, or number of tonsilloliths. However, exploratory subgroup analyses showed that smokers exhibited significantly higher frequencies of unilateral tonsilloliths (*p* = 0.041) and lingual tonsilloliths (*p* = 0.036) than non-smokers. No significant association was observed between sex and the presence or size of tonsilloliths. These findings suggest that smoking may influence the anatomical distribution of tonsilloliths rather than their overall occurrence.

Although smoking does not appear to affect the overall prevalence of tonsilloliths, our findings indicate that unilateral and lingual tonsilloliths are more common in smokers, and the localization distribution is significantly different, with lingual tonsilloliths being more frequent in smokers and palatine tonsilloliths in non-smokers. Cigarette smoke causes chronic inflammation, oxidative stress, and an impaired local immune response in the oropharyngeal mucosa [12]. Histopathological studies have shown that the tonsillar crypt epithelium of smokers exhibits basement membrane disruption, cellular degeneration, and superficial erosions. These findings indicate a loss of mucosal integrity, which is thought to contribute to tonsillolith formation. Such epithelial alterations may facilitate the retention of cellular debris and bacterial colonization within the crypts, thereby promoting conditions favorable for subsequent calcification. One possible explanation for the higher frequency of lingual tonsilloliths among smokers is greater exposure of the lingual tonsillar tissue to cigarette smoke and heat because of its anatomical proximity to the tongue base, potentially resulting in more pronounced local irritation and chronic inflammation. Similarly, the increased frequency of unilateral tonsilloliths in smokers may be interpreted in light of habitual smoking behavior, such as the preferred position of the cigarette within the lips and the resulting direction of smoke flow, which could lead to asymmetric exposure of the tonsillar tissues to tobacco smoke. Alternatively, pre-existing anatomical asymmetries in crypt architecture between the two tonsils may render one side inherently more susceptible to debris retention and calcification, with smoking acting as an aggravating rather than a primary causative factor. Because smoking-side preference, hand dominance, and other behavioral variables were not assessed in the present retrospective study, these explanations remain hypothetical and should be investigated in future well-designed prospective studies.

Our study revealed that the presence and size of tonsilloliths showed a statistically significant relationship with age. The findings of the present study are consistent with the existing literature. Takahashi et al. [3], in their evaluation of computed tomography (CT) images from 2,873 consecutive patients, reported that the prevalence of palatine tonsilloliths increased significantly after the age of 40, with the highest prevalence observed in the 50–69-year age group. Similarly, Oda et al. [13], in their analysis of 482 paired CT and panoramic radiographs, demonstrated a significant correlation between age and the detection of tonsilloliths and reported that the detection rate was significantly higher in individuals older than 40 years than in those younger than 40 years. Likewise, Kalabalık et al. [14], who evaluated 399 CBCT scans from a Turkish population in the Aegean region, found a positive correlation between age and the number of tonsilloliths and showed that tonsillar calcifications were significantly more prevalent in individuals aged 41 years and older, whereas they were significantly less common in those aged 20 years or younger. More recently, Yasan et al. [7] reported that the mean age of patients with tonsilloliths (42.39 ± 16.03 years) was significantly higher than that of patients without tonsilloliths (37.50 ± 15.56 years). Taken together, these consistent findings strongly suggest that tonsillolith formation is a progressive, age-dependent process.

Our analysis showed no statistically significant difference between the sexes in the presence (*p* = 0.515) or size (*p* = 0.443) of tonsilloliths. Although some studies report no significant difference between the sexes [13, 15, 16], other studies indicate that the prevalence significantly differs between the sexes [11, 17]. Tonsilloliths were detected in 31.4% of the males and 27.8% of the females included in our study. These conflicting results support the generally accepted view that the etiology of tonsilloliths is determined primarily by local factors (crypt morphology, debris accumulation, inflammation) rather than biological sex alone.

A further point warranting discussion is the radiographic differential diagnosis of tonsilloliths. Not all radiopaque foci detected in the tonsillar region represent tonsilloliths; phleboliths and calcified carotid plaques may exhibit a similar radiographic appearance on CBCT and should be differentiated based on their characteristic morphology and anatomical location [18]. Rarely, unusually large calcified masses in this region may represent giant tonsilloliths presenting as symptomatic oropharyngeal masses and may be difficult to distinguish clinically from a neoplastic process until histopathological confirmation is obtained [19]. Awareness of these possibilities is particularly important in smokers, who have an increased risk of vascular calcification; therefore, incidental calcifications detected on CBCT in this population should be interpreted with careful radiographic assessment.

This study has several limitations that should be acknowledged. First, it used dental CBCT rather than medical CT. Although CBCT provides high spatial resolution for imaging mineralized structures, its limited soft-tissue contrast resolution may reduce the sensitivity for detecting small or minimally calcified tonsilloliths. Consistent with this, published estimates of tonsillolith prevalence appear to vary not only according to the study population but also according to the imaging modality used. CBCT-based studies have generally reported prevalence rates ranging from 18.6% to 33.2% [2, 14, 16, 20], whereas studies using medical CT have reported prevalence rates between 24.6% and 46.1% [3, 7, 13, 17, 21] (Table 3). Therefore, the overall prevalence observed in our study (29.6%) may, at least in part, reflect the lower detection sensitivity of CBCT compared with medical CT rather than true differences in the underlying population. In addition, because CBCT provides limited soft-tissue contrast [22], precise differentiation between palatine and lingual tonsillar tissues may be less reliable than with medical CT. Furthermore, due to the retrospective design of the present study, it was not possible to evaluate more granular aspects of smoking behavior, such as the side of lip contact, the direction of smoke flow, or the depth of inhalation. Another limitation of the present study is that the laterality of unilateral tonsilloliths (right versus left side) was not recorded. Consequently, no conclusions could be drawn regarding a possible relationship between the preferred position of the cigarette within the lips, the direction of smoke flow, and the side of tonsillolith occurrence. Future prospective studies should systematically document, particularly in patients with unilateral tonsilloliths, the side of lesion involvement (right or left) together with smoking-related behavioral variables, such as the preferred position of the cigarette within the lips and the direction of smoke flow, using patient questionnaires and clinical examinations. This approach may help clarify the potential role of smoking behavior in the lateralized distribution of tonsilloliths. The frequently reported asymmetric distribution of tonsilloliths in the literature [17] suggests that localized factors, such as the direction of smoke flow, may influence debris retention within the tonsillar crypts. However, this hypothesis requires validation through well-designed prospective studies.

**Table 3.** Reported tonsillolith prevalence by imaging modality and population

Study	Modality	Population	*N*	Prevalence
Moshfeghi et al. [20]	CBCT	Iran	290	18.6%
Kalabalık et al. [14]	CBCT	Turkey	399	25.8%
Demiralp et al. [16]	CBCT	Turkey	366	20.4%
Ozdede et al. [2]	CBCT	Turkey	527	33.2%
Fauroux et al. [21]	Medical CT	France	150	24.6%
Yasan et al. [7]	Medical CT	Turkey	3,259	27.6%
Kim et al. [17]	Medical CT	South Korea	3,886	30.3%
Takahashi et al. [3]	Medical CT	Japan	2,873	39.9%
Oda et al. [13]	Medical CT	Japan	482	46.1%

CT, computed tomography; CBCT, cone-beam computed tomography; *N*, number of participants.

## Conclusion

This study found no significant association between smoking status and the overall prevalence, size, or number of tonsilloliths. However, smoking was significantly associated with unilateral involvement and a higher frequency of lingual tonsilloliths, suggesting that local effects of smoking may influence the anatomical distribution of tonsilloliths rather than their overall occurrence. Given the retrospective design and the methodological limitations of the present study, these findings should be interpreted with caution. Future prospective studies incorporating detailed smoking-exposure parameters, potential confounding variables, and multimodal imaging or biological inflammatory markers are warranted to clarify the role of tobacco exposure in the pathogenesis and localization of tonsilloliths.

## Statement of Ethics

The study protocol was submitted to and approved by the Clinical Research Ethics Board of Akdeniz University Faculty of Medicine (Approval No.: 343; approval date: 11.05.2022) and the study was conducted in accordance with the Declaration of Helsinki. This research was conducted as a retrospective cross-sectional study using existing CBCT images and medical records. As no prospective intervention or clinical trial procedures were performed, clinical trial registration was not applicable.

## Conflict of Interest Statement

The authors have no conflicts of interest to declare.

## Funding Sources

This study was not supported by any sponsor or funder.

## Author Contributions

Ali Rasat and Sevcihan Günen Yılmaz conceptualized the study. Ali Rasat performed data curation, formal analysis, and visualization and prepared the original draft of the manuscript. Sevcihan Günen Yılmaz contributed to methodology, supervision, and manuscript review and editing. All authors approved the final manuscript.

## Data Availability

The data that support the findings of this study are available on request from corresponding author.

## References

[1] Parihar A , Shastri A, Jain A, Saxena A, Rawat A, Singh M. “CALCIFIC ISLANDS:” A cone-beam computed tomography review of soft-tissue calcifications in head-and-neck region. J Oral Maxillofac Radiol. 2020;8(2):30.

[2] Ozdede M , Akay G, Karadag O, Peker I. Comparison of panoramic radiography and cone-beam computed tomography for the detection of tonsilloliths. Med Princ Pract. 2020;29(3):279–84. 10.1159/000505436 PMC7315145 31842016

[3] Takahashi A , Sugawara C, Kudoh T, Uchida D, Tamatani T, Nagai H, . Prevalence and imaging characteristics of palatine tonsilloliths detected by CT in 2,873 consecutive patients. The Scientific World J. 2014;2014:1–4. 10.1155/2014/940960 PMC4214167 25389536

[4] Yıldırım D , Bilgir E. Soft tissue calcifications and ossifications in the head and neck Region. J Atatürk Univ Fac Dentistry. 2016;25(13).

[5] Çitir M , Gündüz K. Panoramik radyografide yumuşak doku Kalsifikasyon/ossifikasyonlarının Görülme sıklığı. Selcuk Dental J. 2020;7(2):226–32.

[6] Gurbuz E , Gungor M, Hatipoglu H. Radiographic detection of the relationship between tonsilloliths and dental plaque-related pathologies in a series of digital panoramic radiographs. Med Princ Pract. 2022;31(2):149–55. 10.1159/000521687 PMC9210027 34963117

[7] Yasan H , Sivrice ME, Kumbul YÇ, Büyükçelik B, Kayan M. Is there a relationship between tonsillolith formation and nasal septal deviation or chronic sinusitis, with or without nasal polyps? Turk Arch Otorhinolaryngol. 2025;63(1):16. 10.4274/tao.2025.2024-12-10 PMC12178226 40353758

[8] Radu A , Custura AM. Tonsilloliths: causes, symptoms, and treatment options. MedMater. 2025;5(2):59–64.

[9] Mayr A , Ciper N, Wahl G, Wildenhof J, Frede S, Kirschneck C, . Longitudinal analysis of microcirculatory parameters in gingival tissues after tooth extraction in patients with different risk profiles for wound healing disorders - a pilot study. Clin Oral Investig. 2024;28(6):303. 10.1007/s00784-024-05686-3 PMC11076346 38714559

[10] Silva H . Tobacco use and periodontal disease-the role of microvascular dysfunction. Biology. 2021;10(5):441. 10.3390/biology10050441 PMC8156280 34067557

[11] Takahashi A , Sugawara C, Kudoh K, Yamamura Y, Ohe G, Tamatani T, . Lingual tonsillolith: prevalence and imaging characteristics evaluated on 2244 pairs of panoramic radiographs and CT images. Dentomaxillofac Radiol. 2018;47(1):20170251. 10.1259/dmfr.20170251 PMC5965738 28937280

[12] Cinamon U , Goldfarb A, Marom T. The impact of tobacco smoking upon Chronic/Recurrent Tonsillitis and post tonsillectomy bleeding. Int Arch Otorhinolaryngol. 2017;21(2):165–70. 10.1055/s-0036-1593835 PMC5375702 28382125

[13] Oda M , Kito S, Tanaka T, Nishida I, Awano S, Fujita Y, . Prevalence and imaging characteristics of detectable tonsilloliths on 482 pairs of consecutive CT and panoramic radiographs. BMC Oral Health. 2013;13(1):54. 10.1186/1472-6831-13-54 PMC3852777 24125195

[14] Kalabalık F , Çiftçi C, Aytuğar E. Investigation of the prevalence of tonsillolith in the Aegean Region using cone-beam computed tomography. Kocaeli Üniversitesi Sağlık Bilimleri Dergisi. 2019;5(3):129–33.

[15] Yu CL , Tao LX, Li XH, Yu YQ, Liu B. Prevalence of tonsilloliths and CT diagnosis. Chin J Otorhinolaryngol Head Neck Surg. 2017;52(8):604–7. 10.3760/cma.j.issn.1673-0860.2017.08.009 28822414

[16] Şebnem E , Çakmak K, Bir R, Kemal Ç, Demiralp Ö, Bayrak S. Prevalence and characterıstıcs of Palatıne Tonsıllolıths in cone beam computed tomography images: a retrospectıve Study. Kırıkkale Üniversitesi Tıp Fakültesi Dergisi. 2017;19(2):97–102.

[17] Kim MJ , Kim JE, Huh KH, Yi WJ, Heo MS, Lee SS, . Multidetector computed tomography imaging characteristics of asymptomatic palatine tonsilloliths: a retrospective study on 3886 examinations. Oral Surg Oral Med Oral Pathol Oral Radiol. 2018;125(6):693–8. 10.1016/j.oooo.2018.01.014 29506917

[18] Al-Haj HA , Necker FN, Stadlinger B. Imaging of tonsilloliths: a comparative analysis of representation across different radiological modalities. Case Rep Dent. 2026(1):2026. https://doi:10.1155/crid/6877625 10.1155/crid/6877625 PMC12831118 41585956

[19] Krishnan PS , Bowers EMR, D’Aguillo CM. Giant tonsillolith presenting as an oropharyngeal mass. Otolaryngol Case Rep. 2024;30:100572.

[20] Moshfeghi M , Navabi S, Soltani P, Moaddabi A. Prevalence of tonsillolith on cone beam computed tomography images in patients attending shahid beheshti University of Medical Sciences. Br J Med Med Res. 2017;19(11):1–6.

[21] Fauroux MA , Mas C, Tramini P, Torres JH. Prevalence of palatine tonsilloliths: a retrospective study on 150 consecutive CT examinations. Dentomaxillofac Radiol. 2013;42(7):20120429. 10.1259/dmfr.20120429 PMC3699974 23628744

[22] Suomalainen A , Pakbaznejad Esmaeili E, Robinson S. Dentomaxillofacial imaging with panoramic views and cone beam CT. Insights Imaging. 2015;6(1):1–16. 10.1007/s13244-014-0379-4 PMC4330237 25575868

